# Supplementary figures and images for: Quieting the Storm: Hypoxia as a Strategy to Boost UC-MSC Therapies for Hypoxic-Ischemic Brain Lesions in Neonatal Rats
Source: Stem Cell Rev Rep. 2026 Mar 30;22(4):1974–2000. doi: 10.1007/s12015-026-11089-6 (PMC13100022; doi:10.1007/s12015-026-11089-6)

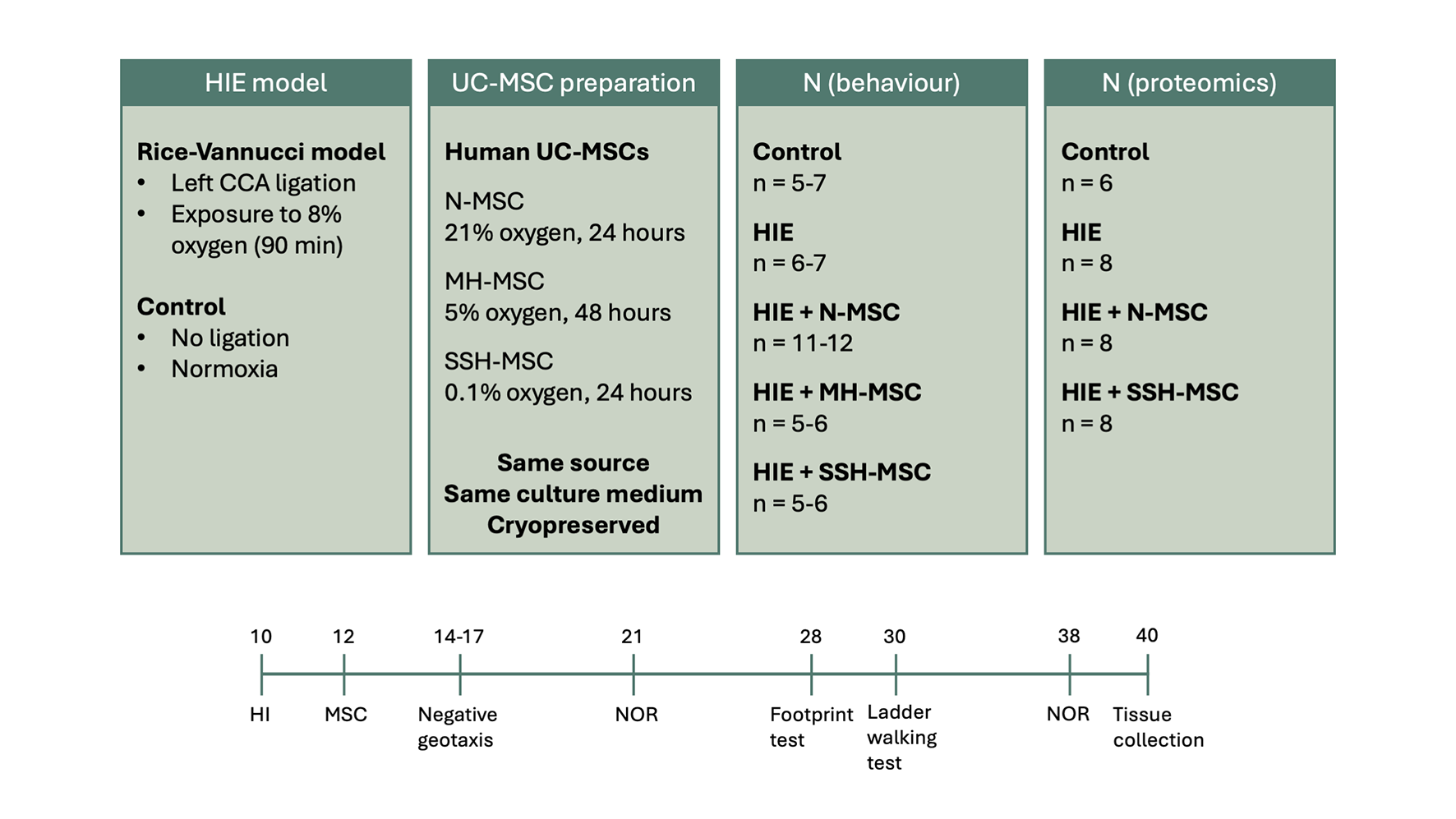

Supplement: Supplementary file 1 — (PNG 243 KB) [file 12015_2026_11089_Fig13_ESM.png]

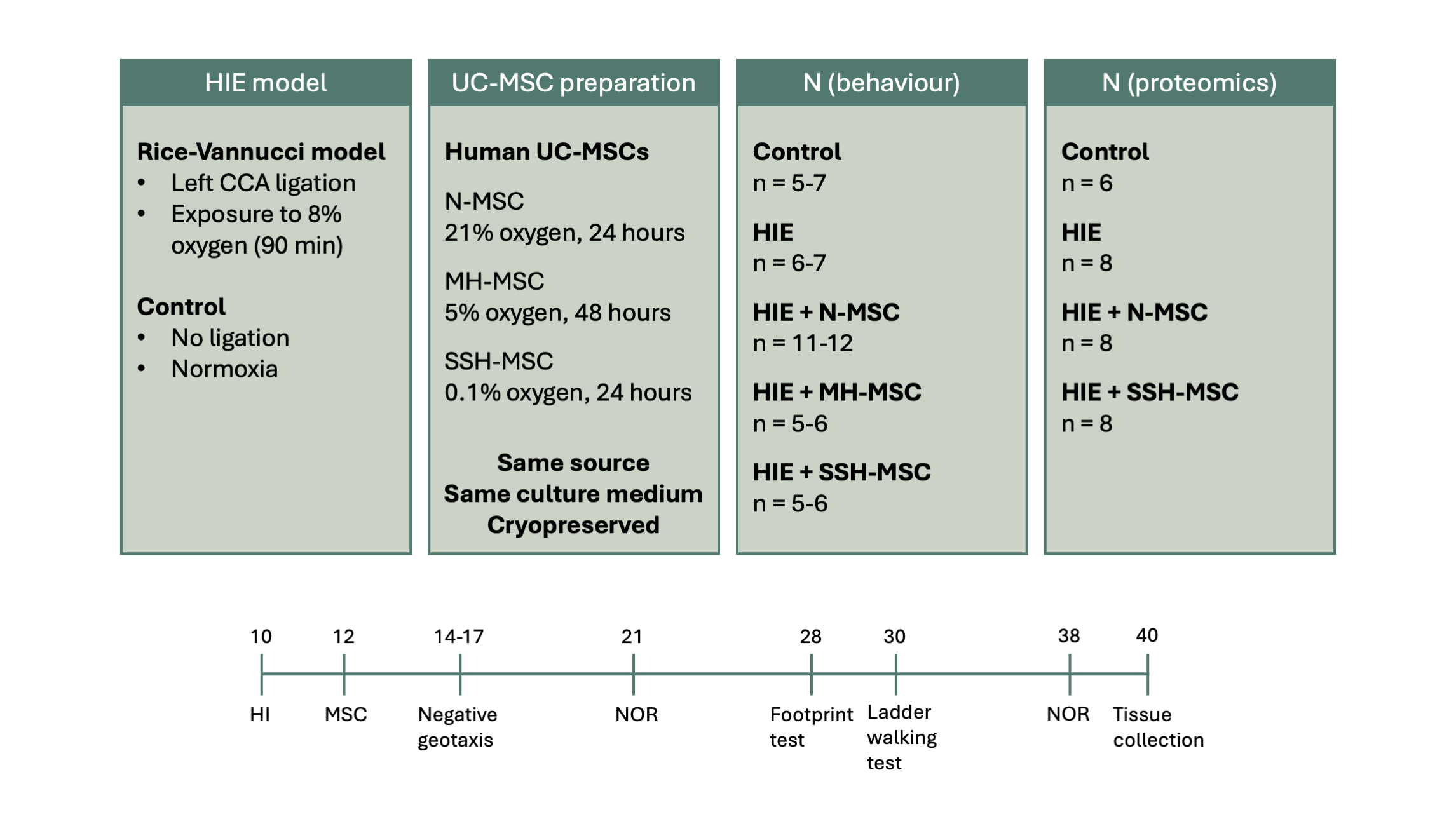

Supplement: Supplementary file 2 — (TIF 8.45 MB) [file 12015_2026_11089_MOESM1_ESM.tiff]

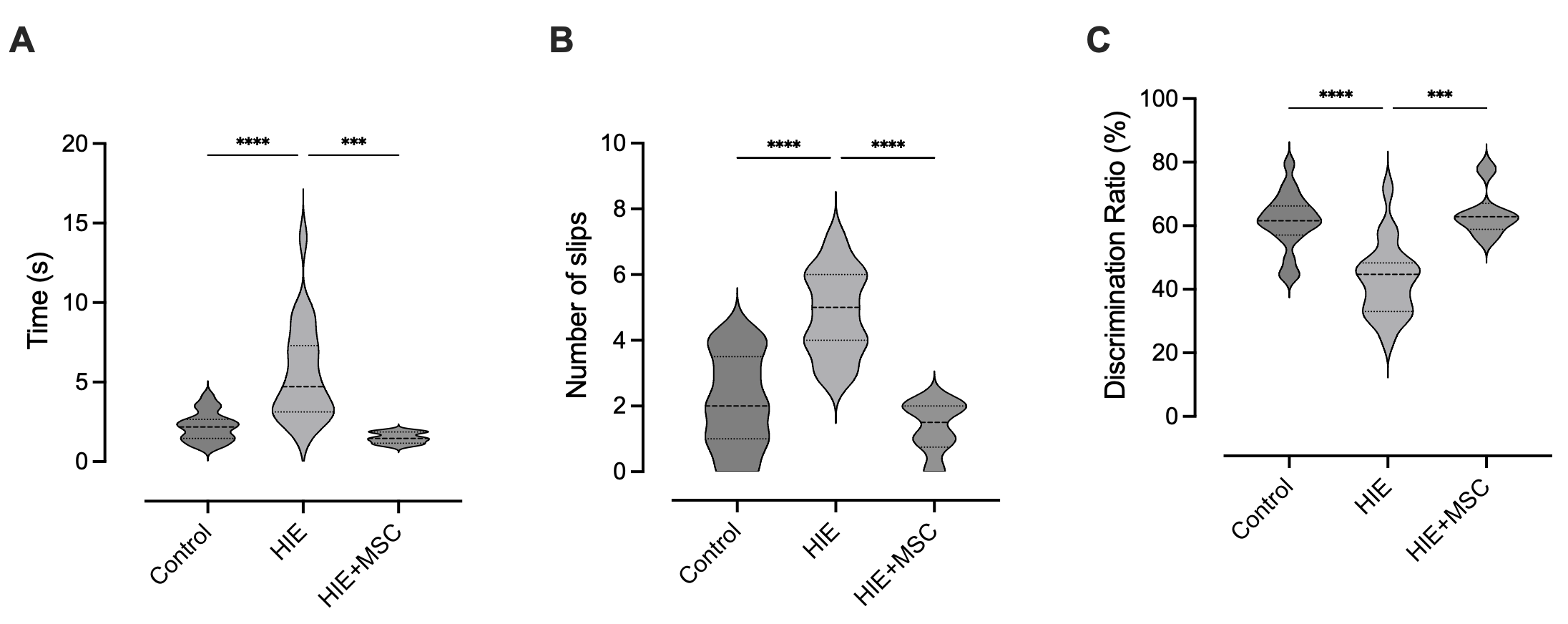

Supplement: Supplementary file 3 — (PNG 186 KB) [file 12015_2026_11089_MOESM2_ESM.png]
