# Supplementary material for: Quieting the Storm: Hypoxia as a Strategy to Boost UC-MSC Therapies for Hypoxic-Ischemic Brain Lesions in Neonatal Rats
Source: Stem Cell Rev Rep. 2026 Mar 30;22(4):1974–2000. doi: 10.1007/s12015-026-11089-6 (PMC13100022; doi:10.1007/s12015-026-11089-6)
Supplement: Supplementary file 6 — Supplementary PDF 6 (PDF 125 KB) [file 12015_2026_11089_MOESM5_ESM.pdf]

Additional Table 1 - Summary of experimental conditions and statistical analysis. The table includes the figure number, experimental condition details, sample size (N), mean values, and standard deviations (SD) for each condition.

Figure	Experimental Condition	N	Mean	SD	N excluded	Reason
Figure 1A P14	Control	7	1.429	0.4016	0	
	HIE	7	4.677	1.47	0	
	HIE+N-MSC	12	3.768	0.6061	0	
	HIE+MH-MSC	6	2.153	1.214	0	
	HIE+SSH-MSC	6	3.235	0.8621	0	
Figure 1A P17	Control	7	1.024	0.4771	0	
	HIE	7	3.319	1.242	0	
	HIE+N-MSC	12	3.847	1.276	0	
	HIE+MH-MSC	6	1.038	0.6201	0	
	HIE+SSH-MSC	6	3.328	0.6911	0	
Figure 1B Overlap	Control	5	1.6	1.517	1	Lack of motivation to walk
	HIE	6	4.667	0.8165	0	
	HIE+N-MSC	11	4.727	1.618	1	Lack of motivation to walk
	HIE+MH-MSC	5	2	0.7071	1	Lack of motivation to walk
	HIE+SSH-MSC	6	5.167	1.602	0	
Figure 1B Drag	Control	5	0.6	0.8944	1	Lack of motivation to walk
	HIE	6	2.333	1.506	0	
	HIE+N-MSC	11	2.909	1.044	1	Lack of motivation to walk
	HIE+MH-MSC	5	0.4	0.5477	1	Lack of motivation to walk
	HIE+SSH-MSC	6	3.833	1.602	0	
Figure 1C	Control	7	0.714	0.756	0	
	HIE	7	4.71	1.38	0	
	HIE+N-MSC	12	4.58	1.38	0	
	HIE+MH-MSC	6	2.5	1.38	0	

	HIE+SSH-MSC	5	3.4	1.67	1	Lack of motivation to cross the ladder
Figure 2 P21	Control	7	68.38	6.961	0	
	HIE	7	39.54	11.39	0	
	HIE+N-MSC	11	42.65	11.96	1	Lack of motivation to explore the objects
	HIE+MH-MSC	4	72.75	12.14	2	Lack of motivation to explore the objects
	HIE+SSH-MSC	6	48.47	9.039	0	
Figure 2 P38	Control	7	63.97	4.262	0	
	HIE	7	38.47	6.383	0	
	HIE+N-MSC	12	44.83	10.96	0	
	HIE+MH-MSC	6	66.32	7.797	0	
	HIE+SSH-MSC	6	46.64	2.667	0	
Figure 12 APOE	Control	6	1.01069	0.021776		
	HIE	8	1.90049	0.69613		
	HIE+N-MSC	8	1.82863	0.597074		
	HIE+SSH-MSC	8	1.23923	0.276125		
Figure 12 AQP4	Control	6	1.0046	0.147508		
	HIE	8	1.75602	0.451723		
	HIE+N-MSC	8	1.81559	0.499213		
	HIE+SSH-MSC	8	1.26957	0.333612		
Figure 12 CLIC	Control	6	1	0.412424		
	HIE	8	1.91783	0.6506		
	HIE+N-MSC	8	1.94846	0.787597		
	HIE+SSH-MSC	8	1.27104	0.423549		
Figure 12 CD44	Control	6	0.87995	0.305941		
	HIE	8	1.90146	0.77436		
	HIE+N-MSC	8	1.73689	0.585075		
	HIE+SSH-MSC	8	1.01898	0.315771		
Figure 12 FAB7	Control	6	1.01172	0.156127		
	HIE	8	2.74003	1.213406		

	HIE+N-MSC	8	2.19416	0.954396		
	HIE+SSH-MSC	8	1.31114	0.367497		
Figure 12 GFAP	Control	6	1.06319	0.321164		
	HIE	8	9.14964	7.296334		
	HIE+N-MSC	8	9.39868	7.117257		
	HIE+SSH-MSC	8	2.58286	1.182972		
Figure 12 SDC4	Control	6	1.00335	0.284811		
	HIE	8	2.13728	0.70914		
	HIE+N-MSC	8	2.02961	0.674365		
	HIE+SSH-MSC	8	1.44531	0.593797		
